# Hybrid Indoor Localization Using IMU Sensors and Smartphone Camera

**DOI:** 10.3390/s19235084

**Published:** 2019-11-21

**Authors:** Alwin Poulose, Dong Seog Han

**Affiliations:** School of Electronics Engineering, Kyungpook National University, 80 Daehak-ro, Buk-gu, Daegu 41566, Korea; alwinpoulosepalatty@knu.ac.kr

**Keywords:** indoor positioning system (IPS), pedestrian dead reckoning (PDR), heading estimation, indoor navigation, IMU sensors, smartphone camera, Kalman filter, sensor fusion, simultaneous localization and mapping (SLAM), ArUco markers

## Abstract

Smartphone camera or inertial measurement unit (IMU) sensor-based systems can be independently used to provide accurate indoor positioning results. However, the accuracy of an IMU-based localization system depends on the magnitude of sensor errors that are caused by external electromagnetic noise or sensor drifts. Smartphone camera based positioning systems depend on the experimental floor map and the camera poses. The challenge in smartphone camera-based localization is that accuracy depends on the rapidness of changes in the user’s direction. In order to minimize the positioning errors in both the smartphone camera and IMU-based localization systems, we propose hybrid systems that combine both the camera-based and IMU sensor-based approaches for indoor localization. In this paper, an indoor experiment scenario is designed to analyse the performance of the IMU-based localization system, smartphone camera-based localization system and the proposed hybrid indoor localization system. The experiment results demonstrate the effectiveness of the proposed hybrid system and the results show that the proposed hybrid system exhibits significant position accuracy when compared to the IMU and smartphone camera-based localization systems. The performance of the proposed hybrid system is analysed in terms of average localization error and probability distributions of localization errors. The experiment results show that the proposed oriented fast rotated binary robust independent elementary features (BRIEF)-simultaneous localization and mapping (ORB-SLAM) with the IMU sensor hybrid system shows a mean localization error of 0.1398 m and the proposed simultaneous localization and mapping by fusion of keypoints and squared planar markers (UcoSLAM) with IMU sensor-based hybrid system has a 0.0690 m mean localization error and are compared with the individual localization systems in terms of mean error, maximum error, minimum error and standard deviation of error.

## 1. Introduction

Indoor localization systems are classified as either building dependent or building independent based on the sensors used for localization [1]. The most common building independent indoor positioning technologies are pedestrian dead reckoning (PDR) systems using inertial measurement unit (IMU) sensors [2,3,4] and image based technologies using cameras [5,6,7]. The IMU sensor used in PDR systems includes the accelerometer, magnetometer and gyroscope sensors and these sensors give user position based on user heading and step length information. In image based indoor positioning, a camera is used for localization and any of monocular, stereo or RGB-D cameras can be used for localization. The camera captures the experiment area and the captured data is fed to image-based localization algorithms. The most popular image-based localization system, which consists of family of algorithms is the simultaneous localization and mapping (SLAM) [8,9]. In SLAM based localization, the system estimates the position or orientation of the camera with respect to its surrounding and maps the environment based on the camera location. Localization using either IMU sensors or a camera system offers some level of accuracy although not the best. The limited level of accuracy in IMU sensors is due to the accumulated errors from accelerometer, drift errors from gyroscope and external magnetic fields that affect the magnetometer. These sensor errors degrade indoor position accuracy, hence the need for compensation.

In this paper, a camera-based system is introduced to the IMU localization system. However, it should be noted that in the camera based localization system, rapid user direction changes affect the effectiveness of the camera pose estimation. In the proposed hybrid systems, the results from the IMU-based system are used to compensate the heading error from the camera-based system. Similarly, the IMU sensor errors are compensated for by utilizing results from the camera-based system. Summarily, in this paper, we propose hybrid indoor localization systems that combine the results from IMU and camera based systems to improve positioning accuracy. Experimental results demonstrate the effect of the proposed hybrid fusion method and the proposed hybrid method reduces the sensor errors for IMU localization and heading error for the camera based localization system. The main contributions of this paper are as follows:

- We implemented an IMU-based indoor localization system. The proposed IMU system uses the accelerometer, gyroscope and magnetometer for position estimation. A pitch based estimator is used for step detection and step length estimation. A sensor fusion algorithm is used for heading estimation. The user position is estimated by using step length and heading information.
- We followed an oriented fast rotated binary robust independent elementary features (ORB)-SLAM algorithm proposed by Mur-Artal et al. [10] for the camera based localization system. The ORB-SLAM uses the same features for tracking, mapping, relocalization and loop closing. This makes the ORB-SLAM system more efficient, simple and reliable as compared to other SLAM techniques.
- We developed a SLAM by fusion of keypoints and squared planar markers (UcoSLAM) algorithm proposed by Munoz-Salinas et al. [11] for the camera based localization system by adding markers to the experiment area. We used Augmented Reality Uco Codes (ArUco) markers for localization and the markers improved the localization accuracy.
- We proposed hybrid indoor localization systems using an IMU sensor and a smartphone camera. The sensor fusion is achieved by a Kalman filter and the proposed systems reduced the IMU sensor errors and heading errors from camera-based localization systems.

The rest of the paper is organized as follows: in Section 2, a review of previous work is discussed. A model for indoor localization using an IMU and a camera is presented in Section 3. The experimental setup and result analysis is given in Section 4. Finally, Section 5 concludes the work and gives future directions.

## 2. Related Work

Indoor localization has been studied in the past and recent times based on different localization techniques and technologies used [12,13,14,15]. In this section, we discuss the existing technologies used for IMU-based localization, camera based localization and various existing hybrid approaches for indoor localization.

The IMU-based indoor localization uses an accelerometer, gyroscope and magnetometer sensors for position estimation and is also known as pedestrian dead reckoning (PDR). Different PDR approaches [16,17] have been proposed for indoor localization and these approaches have a significant role in indoor positioning. The PDR study includes step detection [18,19,20], step length estimation [21,22,23,24,25], heading estimation [26,27,28] and position estimation using step length and heading information. The accuracy of PDR position results depends on the accurate step length estimation and accurate heading estimation. The basic PDR models are explained in [29,30,31,32,33,34]. In these PDR models, different algorithms for indoor localization are explained and their proposed models show significant position accuracy improvements for IMU-based indoor localization. Smartphone IMU-based indoor localization systems are discussed in [35,36,37,38,39]. The smartphone IMU-based PDR systems have many challenges due to the changes in smartphone coordinates. The changes in smartphone coordinates are based on the user movements, hence affecting IMU sensor readings. Several studies have been conducted on the smartphone IMU-based PDR system in indoor environments during the past years [40,41,42]. These proposed smartphone based IMU localization systems achieve accurate position results for indoor applications. However, the proposed PDR systems still exhibit sensor errors which affect the indoor position accuracy. More recent works on IMU-based localization are shown in [43,44,45,46,47]. In these works they reached significant position accuracy levels for indoor localization. From all these studies, it can be seen that IMU sensor-based localization is not free from sensor errors. It should also be noted that indoor position accuracy depends on accurate sensor reading and calibration. In this paper, we used our previous model presented in [48] for IMU sensor-based localization. In [48], we implemented a pitch based step detector, step length estimator and position estimation algorithm for indoor localization. Our proposed IMU-based localization model in [48] reduced the sensor errors and gives significant results for indoor localization.

A camera based localization system has a major role when the experiment area is independent from the building infrastructure. This method is also known as computer vision [49,50,51,52]. In this method, we use a camera and captures the environment in the form of images or video. The video or image data from the camera is used for estimating the position and orientation of an object or device. Camera based localization can be divided based on the markers used in the indoor area. If the localization system depends on the natural landmarks such as corridors, edges, doors, wall, ceiling light etc., it is referred to as markerless localization [53,54,55,56,57]. If we use some special type of markers such as fiducial markers or ArUco markers in the experiment area, then the localization is known as marker based localization [58,59]. The most common technique used in computer vision is SLAM based localization [60,61,62,63,64]. In SLAM based localization, we create a map of the experiment area and at the same time locate the camera position. The SLAM technique is classified as extended Kalman filter (EKF) SLAM [65,66], FastSLAM [67], low dimensionality (L)-SLAM [68], GraphSLAM [69], Occupancy Grid SLAM [70,71,72], distributed particle (DP)-SLAM [73], parallel tracking and mapping (PTAM) [74], stereo parallel tracking and mapping (S-PTAM) [75], dense tracking and mapping (DTAM) [76,77], incremental smoothing and mapping (iSAM) [78], large-scale direct (LSD)-SLAM [79], MonoSLAM [80], collaborative visual SLAM (CoSLAM) [81], SeqSLAM [82], continuous time (CT)-SLAM [83], UcoSLAM [11], RGB-D SLAM [84] and ORB SLAM [85,86,87]. In this paper, we used ORB SLAM and UcoSLAM for camera based localization. The ORB SLAM is a feature based localization system and it operates in real time in indoor environments. The ORB SLAM includes tracking, mapping, relocalization and loop closing. The ORB SLAM achieved significance indoor position accuracy as compared to other state-of-the-art monocular SLAM approaches. To improve the localization accuracy of camera based localization systems, a simple marker–based localization system is introduced in [88]. The localization system proposed in [88] added the markers into the map with a Tf package to the robot operating system (ROS) [89]. A 2D marker based monocular visual-inertial EKF-SLAM system is proposed in [90] and a planar marker based mapping and localization system is explained in [91]. The marker based systems explained have reduced the localization error as compared to markerless localization. However, the marker based localization systems have serious problems such as mapping distortion due to lack of correction, drift error if the markers are lost for a long time and it requires increasing computation resources to handle a multiple number of markers. In this paper, we use 4 ArUco markers for localization and the UcoSLAM based localization approach gives more accurate results as compared to ORB-SLAM localization approach.

To reduce the sensor errors from IMU-based localization and heading errors for the camera-based system, various studies have proposed a hybrid indoor localization approach. In hybrid based localization, we use the IMU data and camera data together for position estimation. The basic models explained in [92,93,94,95,96,97,98,99,100,101] are used for IMU and camera data fusion. In their proposed methods, they combined the IMU data and vision data for better performance. Their experiment results proved that the hybrid indoor localization system has significant positional accuracy for indoor applications. A comparative study of IMU and camera based localization can be seen in [102]. Most recent work on hybrid localization is discussed in [103,104,105,106,107,108,109]. Among these studies, the hybrid indoor localization can be used for better performance and which reduces IMU sensor and camera position errors. In this paper, we use a linear Kalman filter (LKF) for combining IMU sensor and camera position data. The LKF is simple and easy to implement for real time applications as compared to the extended Kalman filter (EKF) [110,111,112], particle filter [113] and unscented Kalman filter (UKF) [114].

## 3. Model for Indoor Localization Using IMU Sensor and Smartphone Camera

**Figure 1:**
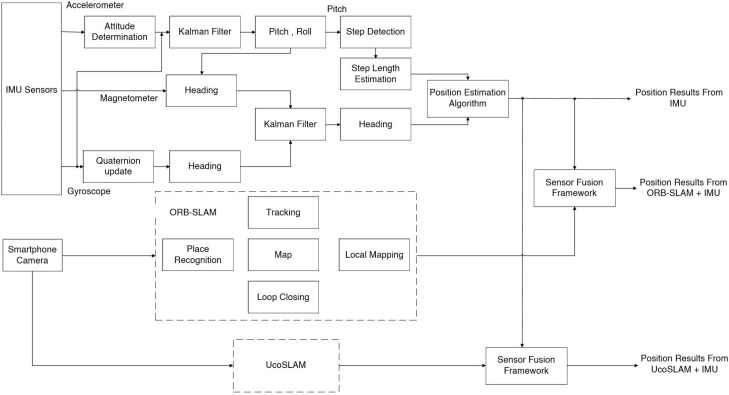
Proposed hybrid indoor localization model using IMU sensor and smartphone camera.

The proposed model for indoor localization using IMU sensor and smartphone camera-based system is shown in Figure 1. In the proposed model, different localization approaches are fused with the sensor fusion frameworks. The proposed model is divided into three steps. Locating user position using IMU sensor is the first step of the proposed model. In IMU-based localization, the position is estimated by step length and heading information. The complementary features of accelerometer, gyroscope and magnetometer sensors are used for position estimation. In the second step, we use a smartphone camera for localization. The smartphone camera captures the experiment area and the captured data used in the ORB SLAM algorithm for position estimation. To improve the camera based localization, we used an UcoSLAM algorithm with ArUco markers. In the last step, we combine the position results from IMU and camera based systems with a Kalman filter. The sensor fusion framework uses a linear Kalman filter for combining position results from IMU and camera.

### 3.1. Indoor Localization Using IMU Sensor

The model presented in our previous work [48] is the same one that we used for IMU-based localization. The proposed model utilizes accelerometer, gyroscope and magnetometer for position estimation. A pitch based estimator is proposed for step detection. A sensor fusion algorithm is used for estimating pitch and roll from accelerometer and gyroscope. The sensor fusion pitch value is used for step detection. The step length is estimated from pitch amplitude. The heading is estimated from gyroscope and magnetometer fusion. Finally, the position is estimated using step length and heading information. For more details on indoor localization using IMU sensor refer to our previous work in [48].

### 3.2. Indoor Localization Using Smartphone Camera

To enhance the indoor position accuracy, we used smartphone camera for localization when the privacy of the user is not a concern. The IMU sensor based localization gives accurate user position results for indoor localization. However, the user position results are not free from sensor error and it is necessary to compensate this sensor error by adding a smartphone camera to the system. In this paper, we used two algorithms for camera based localization. The most common camera based localization algorithm is the ORB-SLAM which gives the user position indoors. However, the keypoints mismatch and camera pose problems, the ORB-SLAM shows heading errors and some user position results are missing during the experiment time. To overcome these problems, we used UcoSLAM algorithm for localization. The UcoSLAM uses special markers called ArUco markers for localization. The ArUco markers solved the camera localization heading problems and improved the position accuracy for localization.

#### 3.2.1. Localization Using ORM-SLAM

The model presented in [10] is used for camera based ORB-SLAM localization. In this localization, we use ORB features [115] instead of scale-invariant feature transform (SIFT) [116] or speeded-up robust feature (SURF) [117,118] for feature matching. The ORB feature matching allows real-time performance without GPUs and it gives best invariance to changes in viewpoint and illumination. The ORB-SLAM consists of tracking, local mapping and loop closing. In the tracking step, we estimate the camera position with every frame and control the new keyframe insertion. We used a motion only bundle adjustment (BA) for optimizing the camera pose using an initial feature matching with previous frame. If the tracking is not done, the place recognition module is used for global relocalization. When the initialization of camera poses and feature map is done, a local map is retrieved using the covisibility graph with keyframes that is used in the system. It then matches with the local map points which are searched by reprojection and finally optimize the camera pose with all matches. The last step of tracking is the new keyframe decision. If the new keyframe is inserted, it is used in the local mapping process. The local mapping uses the new keyframes and performs the local BA to get an optimal reconstruction in the surroundings of the camera pose. Loop closing is the final stage of ORB-SLAM. In loop closing stage, the system searches for loops with every new keyframe. If a loop is identified, a similarity transformation is computed which gives information about the accumulated drift in the loop. If a drift accumulation is detected, then the duplicated points are fused. Finally, to achieve global consistency, a pose graph optimization is performed. For more details on ORB-SLAM refer to [10].

#### 3.2.2. Localization Using UcoSLAM

**Figure 2:**
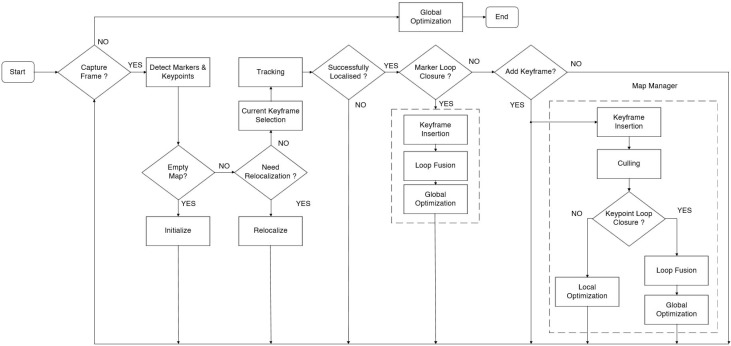
UcoSLAM architecture [11].

The ORB-SLAM approach uses natural landmarks (keypoints) for localization. However, it is unstable over time or insufficient for indoor localization. To improve the indoor localization accuracy, the UcoSLAM method proposed in [11] is used for camera based indoor localization. In UcoSLAM, we use artificial landmarks (ArCuo) in the experiment area for tracking and relocalization. Figure 2 shows the UcoSLAM architecture.

The UcoSLAM follows the same procedure used in the visual SLAM approaches for camera pose estimation. The main difference in the UcoSLAM approach is the combined use of keypoints and ArCuo markers for tracking and relocalization. The UcoSLAM system contains a map of the environment which is created and updated every time a new frame is available to the system. The UcoSLAM starts with map initialization. For map initialization, it uses homography, fundamental matrix (using keypoints) [86] and one or several markers [119]. After the map initialization in UcoSLAM, the system starts tracking or relocalization. If the system determines the camera pose in the last frame, it tries to estimate the current position using the last one as a starting point. In the UcoSLAM system, it uses the reprojection errors of keypoints and marker corners for tracking. For tracking in the UcoSLAM system, a reference frame is selected as map keyframe before tracking and map keyframe contains common matches to the frame analyzed in the previous time instant. After the tracking process, the system searches for the loop closures caused by ArCuo markers. If a loop closure is detected, then the system follows the keyframe insertion, loop fusion and global optimization steps as shown in Figure 2. If the loop closure is not detected, the system uses the map manger block, which runs the culling process when a new keyframe inserted. The culling process helps to maintain the map size manageable by removing the redundant information in the map. After the culling process, the system checks for the keypoint loop closure. If the keypoint loop closure is not detected, the system performs local optimization to integrate the new information. If the keypoint loop closure is detected, the system follows the loop fusion and global optimization steps. If the tracking process in the system failed in the last frame, then the system uses the relocalization mode. In the relocalization mode, it checks the markers already registered on the map. If the relocalization mode is unable to detect the known markers, it uses the bag-of-words (BoW) [120] process. For more details on UcoSLAM refer to [11].

### 3.3. Hybrid Indoor Localization Using IMU Sensor and Smartphone Camera

The objective of hybrid indoor localization is to improve the indoor position accuracy by reducing the IMU and camera sensor errors. For combining IMU localization results with camera based localization results, we used a linear Kalman filter (LKF) instead of other sensor fusion frameworks. The LKF is computationally light and we tackle the problem in a linear perspective. The model presented in [121] is used for sensor fusion frameworks. The system with controlled input and noise is given as

(1) $$X_{g}\left(k\right)=AX_{g}\left(k-1\right)+BU\left(k-1\right)+\Gamma W\left(k-1\right)$$

(2) $$A=\left[\begin{array}{ccc} F & 0_{3\times 3} & 0_{3\times 3}\\ 0_{3\times 3} & F & 0_{3\times 3}\\ 0_{3\times 3} & 0_{3\times 3} & F \end{array}\right]$$

(3) $$B=\left[\begin{array}{ccc} G & 0_{3\times 3} & 0_{3\times 3}\\ 0_{3\times 3} & G & 0_{3\times 3}\\ 0_{3\times 3} & 0_{3\times 3} & G \end{array}\right]$$

(4) $$U\left(k-1\right)=a^{n}\left(k-1\right)$$

with $F=\left[\begin{array}{ccc} 1 & t & -t/2\\ 0 & t & -t\\ 0 & 0 & 1 \end{array}\right]$, $G=\left[\begin{array}{c} t^{2}/2\\ t\\ 0 \end{array}\right]$, where *k* is the variable used for the recursive execution of Kalman filter, *t* is the sample period, *A* is the state-transition matrix, *B* is the controlled input matrix, $0_{3\times 3}$ is the zero matrix, $U\left(k-1\right)$ is the controlled input, Γ is the noise matrix and W(k−1) is the Gaussian white noise with variance Q(k−1). The state vector $X_{g}\left(k\right)$ includes the position results from the IMU sensor. The measurement model of ORB-SLAM + IMU is the camera position $\;Pc\;\left(k\right)=\;\left(P_{x}^{C}\left(k\right),\;P_{y}^{C}\left(k\right)\right)$ and the measurement function is

(5) $$z_{1}\left(k\right)={\left[\begin{array}{ccc} P_{x}^{C}\left(k\right) & 0 & \begin{array}{ccc} 0 & P_{y}^{C}\left(k\right) & \begin{array}{cc} 0 & 0 \end{array} \end{array} \end{array}\right]}^{T}=H_{1}X_{1}\left(k\right)+n_{1}\left(k\right)$$

where $H_{1}=diag\left(1,0,0,1,0,0\right)$, $X_{1}\left(k\right)$ is the state vector of ORB-SLAM + IMU fusion, $n_{1}\left(k\right)$ is the measurement noise with covariance matrix $R_{1}\left(k\right)$. The measurement of UcoSLAM + IMU is the camera position $\;Pu\;\left(k\right)=\;\left({\;P}_{x}^{u}\left(k\right),\;P_{y}^{u}\left(k\right)\right)$ and the measurement function is

(6) $$z_{2}\left(k\right)={\left[\begin{array}{ccc} P_{x}^{u}\left(k\right) & 0 & \begin{array}{ccc} 0 & P_{y}^{u}\left(k\right) & \begin{array}{cc} 0 & 0 \end{array} \end{array} \end{array}\right]}^{T}=H_{2}X_{2}\left(k\right)+n_{2}\left(k\right)$$

where $H_{2}=diag\left(1,0,0,1,0,0\right)$, $X_{2}\left(k\right)$ is the state vector of UcoSLAM + IMU fusion, $n_{2}\left(k\right)$ is the measurement noise with covariance matrix $R_{2}\left(k\right)$. The sensor fusion algorithm uses linear Kalman filter since the state and measurement functions are both linear. The Kalman filter consists of two processes, predicting and updating [121].

**Predicting:**

(7) $${\hat{X}}_{i}\left(k,k-1\right)=A{\hat{X}}_{i}\left(k-1\right)+BU\left(k-1\right)$$

(8) $${\hat{P}}_{i}\left(k,k-1\right)=A{\hat{P}}_{i}\left(k-1\right)A^{T}+Q_{i}\left(k-1\right)$$

**Updating:**

(9) $$K_{i}\left(k\right)={\hat{P}}_{i}\left(k,k-1\right)H_{i}^{T}{\left(H_{i}{\hat{P}}_{i}\left(k,k-1\right)H_{i}^{T}+R_{i}\left(k\right)\right)}^{-1}$$

(10) $${\hat{X}}_{i}\left(k\right)={\hat{X}}_{i}\left(k,k-1\right)+K_{i}\left(k\right)\left(z_{i}\left(k\right)-H_{i}{\hat{X}}_{i}\left(k,k-1\right)\right)$$

(11) $$P_{i}\left(k\right)=\left(I-K_{i}\left(k\right)H_{i}\right){\hat{P}}_{i}\left(k,k-1\right)$$

The variables used in the LKF algorithm are summarized in Table 1.

## 4. Experiment and Result Analysis

**Figure 3:**
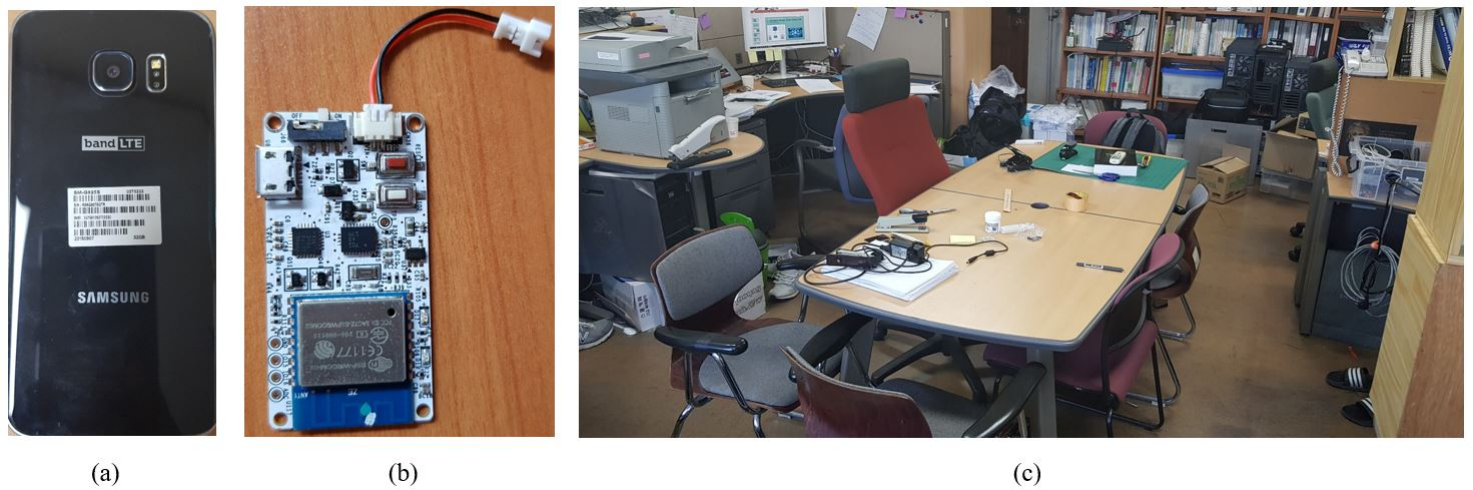
Experiment setup. (**a**) Smartphone. (**b**) IMU sensor. (**c**) Experiment area.

To evaluate the performance and accuracy of the proposed hybrid indoor localization systems, we considered an experiment scenario shown in Figure 3. The data from IMU and camera were collected at the fifth floor of IT building 1, Kyungpook National University, South Korea. During data collection, a user of age 27 and height 172 cm held a smartphone and the IMU sensor in his hand and walked around the table as shown in Figure 3c.

**Figure 4:**
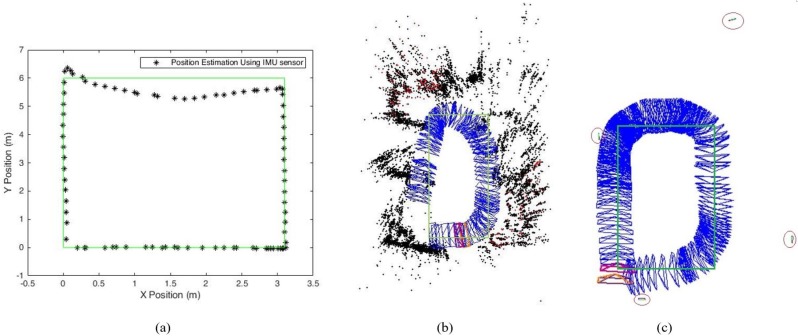
Indoor localization. (**a**) IMU-based localization. (**b**) ORB-SLAM localization. (**c**) UcoSLAM localization.

The experiment was conducted using an Android 5.0.2 Lollipop platform (Google, Mountain View, CA, USA) on the Samsung Galaxy S6 edge smartphone with Exynos 7420 processor and 3 GB ram. A Biscuit™ Programmable Wi-Fi 9-Axis absolute orientation sensor is used for IMU localization. To reduce the computational complexity and delay problems from the proposed algorithm, we carried out the proposed algorithm in an external server computer instead of smartphone to estimate the final user position. The length of the experiment area is 3.1 m and the width is 6 m. The experiment is carried out strictly along the reference path. The IMU sensor and smartphone camera data are collected during the user motions in the reference path. The localization algorithms use the collected data and estimate the current user position. For ground truth value estimation, the starting position of the user is assumed as zero and manually measured the coordinates of the reference path. To analyze the performance of the proposed system, we compared the estimated position results from the localization systems with ground truth values and estimated the user position error in terms of meters. Figure 4 shows the experimental results from IMU-based localization and camera based localization approaches.

From Figure 4, the localization results show that the ArUco markers improved the localization accuracy as compared to the IMU and ORB-SLAM based localization approaches. In IMU-based localization, the accuracy of localization depends on the IMU sensor errors and the position estimation results from IMU approach is not accurate. In the case of ORB-SLAM localization approach, some user points are missing due to the absence of keypoints in the experiment area and the heading error from ORB-SLAM approach is higher than IMU-based localization. To improve the localization accuracy of ORB-SLAM approach, we used an UcoSLAM approach with ArUco markers and the ArUco markers reduced the heading error. The red circles in Figure 4c indicate the ArUco markers used for UcoSLAM localization.

**Figure 5:**
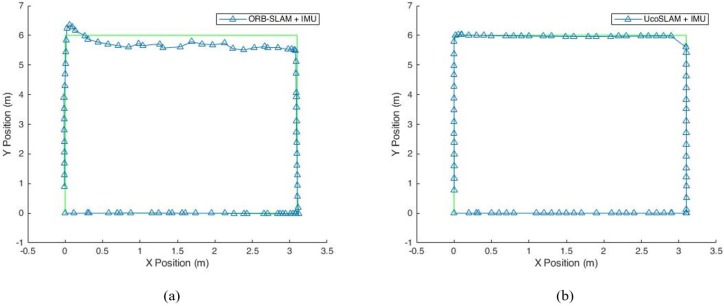
Proposed hybrid systems. (**a**) ORB-SLAM + IMU. (**b**) UcoSLAM + IMU.

To improve the indoor localization accuracy from IMU-based and camera based localization systems, we proposed a hybrid indoor localization system using IMU and camera features together and the estimated position results from proposed hybrid systems are shown in Figure 5.

From Figure 5, it can be seen that the proposed hybrid localization systems remove the effect of IMU sensor errors. The IMU-based localization exhibits accumulated error from the accelerometer and drift error from the gyroscope. The camera based localization cannot estimate the position accurately when user changes direction. The proposed ORB-SLAM + IMU hybrid system overcomes the challenges of IMU and camera based systems. The proposed UcoSLAM + IMU based hybrid system is free from marker based localization problems. When the markers are not detected for a long time, the proposed UcoSLAM uses the IMU position results for localization. The mapping distortion and drift errors in the UcoSLAM are overcome by the effective utilization of the IMU position results. The IMU sensor errors are also reduced by the ArUco markers in the UcoSLAM and the proposed UcoSLAM + IMU outperforms conventional hybrid localization systems and gives high position accuracy for indoor localization.

**Figure 6:**
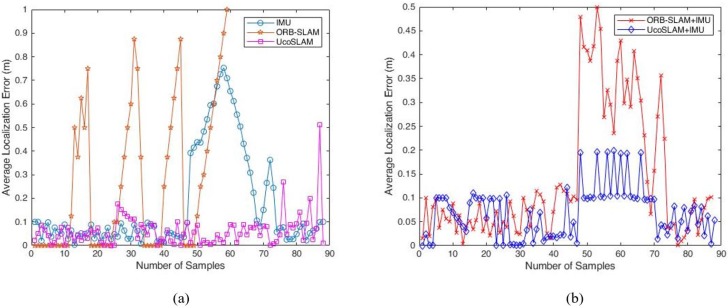
Average localization error. (**a**) IMU-based localization, camera based localization (**b**) Proposed hybrid localization systems.

The accuracy of the proposed hybrid localization systems is evaluated using the average localization error and probability distribution function of localization errors. The average localization error (E) is defined as

(12) $$E=\sum_{i=1}^{L}{\left({\left(x_{i}^{true}-x_{i}^{est}\right)}^{2}+{\left(y_{i}^{true}-y_{i}^{est}\right)}^{2}\right)}^{0.5}$$

where $\left(x_{i}^{true},y_{i}^{true}\right)$ is the actual user position and $\left(x_{i}^{est},y_{i}^{est}\right)$ represents the estimated coordinate of unknown user position calculated by localization methods. *L* is the total number of data samples used for the localization. The average localization error results of IMU-based localization, camera based localization and proposed hybrid localization systems are shown in Figure 6.

Figure 6a shows the average localization error results from IMU, ORB-SLAM and UcoSLAM based localization approaches. The maximum error from IMU-based localization approach is 0.7528 m when compared to the ground truth values. The ORB-SLAM localization approach gives 1 m localization error when compared to the reference path. The UcoSLAM localization approach shows a maximum of 0.5120 m when compared to actual values. Figure 6b shows the average localization error results from the proposed hybrid localization systems. The proposed hybrid systems show reasonable localization accuracy when compared to the true position values. From the average localization error results, the proposed hybrid localization approaches improved the localization accuracy when compared to the independent localization approaches. Table 2 shows the performance analysis of different localization approaches.

**Figure 7:**
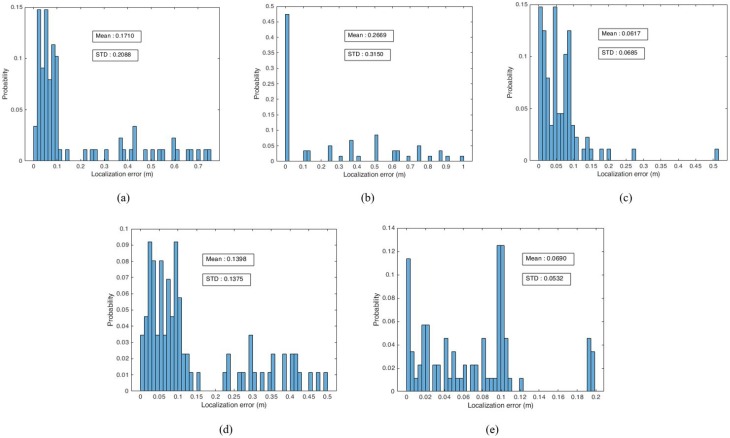
Probability distribution of localization errors. (**a**) IMU-based localization. (**b**) ORB-SLAM localization. (**c**) UcoSLAM localization. (**d**) ORB-SLAM + IMU. (**e**) UcoSLAM + IMU.

From Table 2, the proposed hybrid localization systems have less localization errors as compared to the IMU and camera based localization approaches. The proposed hybrid localization gives high position accuracy as compared to other localization approaches and the accuracy of proposed hybrid approach is analyzed by probability distributions of localization errors. Figure 7 shows the probability distributions of localization errors of IMU-based localization, camera based localization and proposed hybrid localization approaches.

From Figure 7, it is clear that the proposed hybrid localization approach reduces the position errors and gives better performance when compared to the IMU and camera-based localization approaches. The localization system based on ORB-SLAM approach has high possibilities for zero localization error as compared to other localization systems. However, the system cannot estimate all user positions due to the lack of keypoints in the experiment area. The UcoSLAM system has less mean localization error than IMU and ORB-SLAM based systems. The IMU-based localization gives better performance compared to camera-based localization in terms of heading errors. The error analysis shows that the camera-based localization approach is affected by camera pose errors and hence degrades the indoor position accuracy. From all this experiment result analysis, the proposed hybrid localization approach gives reasonable position accuracy for indoor applications.

## 5. Conclusions

This paper presented hybrid indoor localization system using IMU sensor and smartphone camera. The IMU sensor errors are reduced by smartphone camera pose and the heading errors from camera-based localization is overcome by the IMU localization results. The proposed hybrid method show better positioning results compared to the individual localization method. We proposed a Kalman filter based sensor fusion framework for hybrid localization approaches which enhanced the results of the proposed methods. To improve the indoor position accuracy, the authors intend to utilize optical flow and ultrasonic sensors in the proposed system in future work.

## Figures and Tables

**Table 1:** Variables used in the LKF algorithm.

${\hat{X}}_{i}$	Estimated State Vector
$P_{i}\left(k\right)$	Estimated state covariance
$Q_{i}\left(k-1\right)$	Process noise
$R_{i}\left(k\right)$	Measurement noise covariance matrix
$K_{i}\left(k\right)$	Kalman gain matrix
$z_{i}\left(k\right)$	Measurement
*A*	State-transition matrixt
*B*	Controlled input matrix
*H*	Observation model
${\hat{X}}_{i}\left(k,k-1\right),{\hat{P}}_{i}\left(k,k-1\right)$	For internal computation

**Table 2:** Performance of different localization approaches.

Localization Method	Mean Error (m)	Max. Error (m)	Min. Error (m)	Standard Deviation of Error (m)
IMU	0.1710	0.7528	0.0031	0.2088
ORB-SLAM	0.2669	1	0	0.3150
UcoSLAM	0.0617	0.5120	0	0.0685
ORB-SLAM + IMU	0.1398	0.4996	0.0011	0.1375
UcoSLAM + IMU	0.0690	0.1985	0	0.0532
